# Berberine Encapsulated Lecithin–Chitosan Nanoparticles as Innovative Wound Healing Agent in Type II Diabetes

**DOI:** 10.3390/pharmaceutics13081197

**Published:** 2021-08-04

**Authors:** Dibya Sundar Panda, Hussein M. Eid, Mohammed H. Elkomy, Ahmed Khames, Randa M. Hassan, Fatma I. Abo El-Ela, Heba A. Yassin

**Affiliations:** 1 Department of Pharmaceutics, College of Pharmacy, Jouf University, Sakaka 72388, Saudi Arabia; dspanda@ju.edu.sa; 2 Department of Pharmaceutics and Industrial Pharmacy, Faculty of Pharmacy, Beni-Suef University, Beni-Suef 62511, Egypt; hussien.eid@pharm.bsu.edu.eg; 3 Department of Pharmaceutics and Industrial Pharmacy, College of Pharmacy, Taif University, P.O. Box 11099, Taif 21944, Saudi Arabia; dr.akhamies@gmail.com; 4 Department of Cytology and Histology, Faculty of Veterinary Medicine, Beni-Suef University, Beni-Suef 62511, Egypt; randa.abdelgawad@vet.bsu.edu.eg; 5 Department of Pharmacology, Faculty of Veterinary Medicine, Beni-Suef University, Beni-Suef 62511, Egypt; fatma.aboel3la@vet.bsu.edu.eg; 6 Pharmaceutics Department, Faculty of Pharmacy, AlSalam University, Tanta 31527, Egypt; drhobaa48@gmail.com

**Keywords:** berberine, wound healing, lecithin, chitosan, diabetes mellitus, nanotechnology, controlled drug delivery, streptozocin

## Abstract

The aim of this research is to formulate a lecithin–chitosan based nanoparticulate system loaded with berberine (BER-LC-CTS-NPs) that could be integrated into a topically applied formulation and assessed for healing wounds in a diabetic animal model. In order to formulate BER-LC-CTS-NPs, soybean lecithin, isopropyl myristate, and berberine dispersed in ethanolic solution were added into an aqueous solution of chitosan dropwise with sonication. We assessed the influence of lecithin amount, chitosan amount, and isopropyl myristate concentration on particle diameter, zeta potential, and entrapment and employed a Box–Behnken statistical design. The resulting optimized BER-LC-CTS-NPs had a mean size of 168.4 nm, a surface charge of 33.1 mV, and entrapment of 82.3%. The optimized BER-LC-CTS-NPs showed a sustained in vitro release profile. Furthermore, the potential of the optimized BER-LC-CTS-NPs integrated into a topical gel formulation for wound healing in streptozocin-induced diabetic rats was assessed. Our findings show that combining chitosan and berberine in the nanoparticles produces a synergistic effect when it comes to wound healing. The optimized nanoparticulate system works by reducing inflammation, inducing blood vessels and fibroblast proliferation, and promoting mature collagen fibers deposition. Based on the experimental results, lecithin–chitosan nanoparticles loaded with berberine have evolved as a promising strategy for accelerating wound the healing process in diabetic patients. However, the clinical merits of the developed system need to be investigated in diabetic patients.

## 1. Introduction

Chronic non-healing wounds are a significant clinical burden in type 2 diabetic patients, which cause lower mobility and a diminished quality of life, and sometimes results in peripheral limb amputations. Wounds are either resistant to medical treatment or heal very slowly [1]. Wound healing is a natural biological restoration mechanism that uses spontaneous growth and skin tissue regeneration to restore the structural integrity of injured skin. This normally happens through a series of overlapping and interrelated mechanisms such as hemostasis, inflammation, angiogenesis, a proliferation of fibroblasts, and tissue remodeling [2]. Furthermore, the proliferation of keratinocytes is an important mechanism in wound healing [3]. Several cellular and matrix-building processes collaborate during wound recovery to restore the injured site’s damaged integrity [4]. However, even minor injuries to certain parenchymal cells and stromal structure in diabetic patients may result in chronic wounds that impede the regeneration of tissue structure and function [5]. These chronic wounds are also vulnerable to recurring infections, excessive inflammation, and epidermal or dermal cell inability to respond to stimuli [6,7]. Furthermore, abnormalities such as overdeposition of the connective tissue (fibrosis), immunopathy, neuropathy, vasculopathy, growth factor deficiency, and bacterial biofilm are other reasons that cause further delays in diabetic wound healing [8,9]. The existence of Staphylococcus aureus methicillin resistance (MRSA) can complicate the situation further since it is resistant to various antibiotics [10].

Berberine (BER) is an alkaloid of quaternary ammonium salt isolated from amurense rhizome phellodendron. BER is well known for inhibiting Gram-positive and Gram-negative bacteria [11,12], and at the same time, it is stated to have the effect of reducing glucose levels [13]. Interestingly, Zhang et al. reported that BER activates silent information regulator 1 (Sirt1) that results in increased CD31, SMA, and F VEGF expression while inhibiting TNF-a, IL-6, and NF-kB expression, which was beneficial for the healing process in diabetic rats [14]. Sirt1 is a nicotinamide adenine dinucleotide (NAD+)-dependent type III histone deacetylase that is linked to inflammation inhibition, antioxidant stress, and cell ageing [15]. Sirt1 has been shown in experiments to regulate the expression of inflammatory factors and then involve the immune system in the mechanism of disease onset and progression [16,17]. 

Biopolymers such as chitosan (CTS), the deacetylated form of chitin, are known to play a beneficial role in hemostasis as well as in tissue regeneration processes [18,19], prompting the development of fibroblasts [20], and possess activity against fungi and microbes [21,22]. Besides, CTS is a polymer that is biocompatible and biodegradable [23] demonstrating mucoadhesive characteristics [24]. It is apparent that combining BER and CTS will constitute an added value for accelerating the wound healing process in diabetic patients.

Hence, in the current research, we designed BER loaded lecithin (LC) –chitosan (CTS) nanoparticles (BER-LC-CTS-NPs) to be evaluated as a potential agent for accelerating the healing process in rats with streptozocin (STZ)-induced diabetes. This is the first study we are aware of that looks at the benefits of combining BER (as active agent) and CTS (as adjuvant) for wound healing. Size, surface charge, entrapment efficiency, in vitro release, and morphological analysis were all conducted on the BER-loaded nanoparticles. In diabetic rats, in vivo experiments were used to assess the effectiveness of BER-LC-CTS-NPs in the wound healing process.

## 2. Materials and Methods

### 2.1. Materials

Berberine chloride (BER), soybean lecithin (l-a phosphatidylcholine), chitosan (MW: 150 kDa), streptozocin, and dialysis bags (MW cut off: 12 kDa) were supplied from Sigma-Aldrich Chemical Co. (St. Louis, MO, USA). Isopropyl myristate (IPM), potassium dihydrogen orthophosphate, chloroform (HPLC grade), disodium hydrogen orthophosphate, Carbopol 974 NF were supplied from El-Nasr pharmaceutical chemical company (Cairo, Egypt). Other reagents and chemicals utilized in this research were of analytical grade.

### 2.2. Methods 

#### 2.2.1. Design of Experiments and Optimization

The optimization analysis was conducted using Design-Expert software (Version 12.0.3.0, Stat-Ease Inc., Minneapolis, MN, USA) and a Box–Behnken statistical design (BBD) with three variables, three levels, and 17 runs. Based on preliminary tests, the independent variables LC amount (mg) (X_1_), CTS amount (mg) (X_2_), and IPM concentration (% *w*/*v*) (X_3_) were picked and set at low, medium, and high levels. Table 1 summarizes the coded values of different variables. According to the BBD, seventeen formulations were prepared and characterized for particle size (PS: Y1), zeta potential (ZP: Y2), and entrapment efficiency (EE %: Y3), which were selected as response parameters (Table 2). In this design, the main and interaction effects of the independent variables on the formulation aspects are sorted. In this analysis, the objective function was chosen to boost ZP and EE while decreasing PS. In cases with three or four independent variables, the BBD was selected because it needs fewer runs than a central composite design [25]. The polynomial equations generated by the Design-Expert program were validated using the ANOVA test. All collected responses were conducted simultaneously by linear (first), second and quadratic models. Various feasibilities in the experimental field were performed to find the compositions of the optimized BER-LC-CTS-NP formulations. 

Using plot3D R-package, 3D response surface plots were generated [26]. Five optimal checkpoint formulations were selected to check the selected experimental domain. The respective experimental and expected values were quantitatively matched for the response variables.

#### 2.2.2. Preparation of BER-LC-CTS-NPs

BER-LC-CTS-NPs were formulated with minor modifications to previously reported methods [27,28]. Accurately weighed amounts (100, 150, and 200 mg) of LC and 10 mg BER were dissolved in 96% ethanol (4 mL) containing IPM (1%, 2%, and 3% *w*/*v*) to attain different LC concentrations (2.5%, 3.75% and 5% (*w*/*v*)). Likewise, CTS was dissolved in 1% aqueous acetic acid solution (*v/v*) to obtain CTS solution (1%, *w*/*v*). Aqueous acetic acid solution was added to appropriate volumes (1 mL, 2.5 mL, and 4 mL) of 1% CTS solutions (*w/v*) (to obtain 46 mL of diluted CTS solution). BER-LC-CTS-NPs suspensions were synthesized by injecting (40 mL/min injection rate) the LC ethanolic BER solution (4 mL), using 5 mL capacity syringe with a 27-gauge needle, into the diluted CTS solution (46 mL) on a magnetic stirrer (1000 rpm). LC/CTS weight by weight was present in ratios of 10:1, 4:1, 2.5:1, 15:1, 6:1, 3.75:1, 20:1, 8:1, and 5:1 in the end suspension of LC-CTS-NPs and the BER content was 0.02% *w*/*v*. Empty nanoparticles (LC-CTS-NPs) were formulated in the same way with no BER added to the alcoholic LC solution. 

#### 2.2.3. Characterization and Optimization of BER-LC-CTS-NPs

##### Study of Size and Surface Charge of the Nanoparticles

The mean BER-LC-CTS-NPs diameter, given as z-average, was assessed by dynamic light scattering technique in the Zetasizer Nano ZS (Malvern Instruments, Malvern, UK). The assessments were conducted at 25 °C. The freshly prepared BER-LC-CTS-NPs surface charge (given as zeta potential) was measured via laser Doppler micro-electrophoresis technique in Zetasizer Nano ZS (Malvern Instruments, Malvern, UK). 

##### BER Entrapment

The entrapment of BER in LC-CTS-NPs was indirectly determined by subtracting free BER present in the supernatant from the total amount of BER currently used during the formulation (10 mg). In a cooling centrifuge (SIGMA 3–30 K, Germany), BER-LC-CTS-NPs suspension was mounted for 3 h at 4 °C at 14,000 rpm to isolate the supernatant with the free BER [29]. The supernatant was then diluted and analyzed for BER concentration spectrophotometrically (Jasco V-530, Japan) at λmax 345 nm with reference to the developed phosphate-buffered solution calibration curve (pH 7.4) over a 20 to 120 μg concentration range (R^2^ = 0.999). BER entrapment was measured using the following equation

(1) $$\mathrm{EE}\%=\frac{\mathrm{Total}\;\mathrm{amount}\;\mathrm{of}\;\mathrm{BER}\;-\;\mathrm{free}\;\mathrm{BER}}{\mathrm{Total}\;\mathrm{amount}\;\mathrm{of}\;\mathrm{BER}}\times 100$$

### 2.3. Characterization of the Optimized BER-LC-CTS-NPs 

#### 2.3.1. In Vitro Release Study of BER 

The in vitro release experiment was performed with Franz vertical diffusion cells of 5 cm^2^ diffusion region. The donor compartment retained a certain volume of the optimized BER-LC-CTS-NPs formulations (equivalent to 3 mg BER). Fifty milliliters of phosphate-buffered solution (PBS, pH 5.5) were added to the receptor compartment. The rotation speed was 100 rpm and the temperature was 32 ± 0.5 °C [30]. A pre-soaked dialysis membrane was used to separate the donor compartment from the receiver compartment [29]. At set time intervals, a sample of 1 mL was collected and an equivalent quantity of fresh PBS was subsequently applied to the receptor compartment to maintain a fixed volume. The collected samples filtered and spectrophotometrically measured at λmax 345 nm and BER release percentage was determined according to the following equation

(2) $$\mathrm{Drug}\;\mathrm{release}\;\%=\frac{Q_{i}}{Q_{r}}\times 100\;$$

where Q_i_ and Q_r_ represent the quantity of cumulative BER released at *t*-time and the original quantity of BER in BER-LC-CTS-NPs (3 mg), respectively.

#### 2.3.2. The Morphology and pH of the Optimized BER-LC-CTS-NPs

The morphology of the optimized BER-LC-CTS-NPs formulation was studied using transmission electron microscopy (TEM). Briefly, the optimized BER-LC-CTS-NPs suspension was sonicated 10 min before grid preparation. On a clean parafilm, a single copper grid of 300 meshes with type-B carbon support film was placed. On the grid, one drop of sonicated nanosuspension was deposited and allowed to settle for 10 min, then allowed to dry. Using phosphotungstic acid (1% *w*/*v*), the samples were treated as a negative stain and left two minutes for sufficient stain absorption, and excess liquid was collected via filter paper. TEM (Jeol, Tokyo, Japan) was allowed to analyze the samples by operating at a voltage acceleration of 80 kV [31]. The pH of the optimized BER-LC-CTS-NPs was evaluated using a calibrated pH meter (Jenway, London, UK).

#### 2.3.3. Stability Study of the Optimized BER-LC-CTS-NPs

For three months, the optimized BER-LC-CTS-NPs formulation was kept in a glass vial at 4 °C. After preparation, samples from the optimal formulation were collected, and then, after 30, 60, and 90 days of storage, they were analyzed for particle diameter and entrapment [32].

#### 2.3.4. Formulation of Topical BER-LC-CTS-NPs Based Gel

The optimized BER-LC-CTS-NPs formulation, free BER solution, and LC-CTS-NPs (empty nanoparticles) were mixed with Carbopol 974 NF polymer to make a gel according to the method previously reported [32]. Carbopol 974 NF (2%, *w*/*w*) and preservatives (0.01% propylparaben and 0.1% methylparaben) were sprinkled in water and stirred for two to three hours. BER-LC-CTS-NPs (equal to 0.5% *w*/*w* of BER) were then added to the gel base, followed by 1 h stirring. Using Triethanolamine, the pH was changed to 6.0 ± 0.05 to achieve a sufficient strength of BER-LC-CTS-NP gel for topical use. The homogeneity, spreadability, clarity, and proper rheological characteristics of the produced gels were all evaluated. The characterization of the produced gels has been discussed previously [33].

### 2.4. In Vivo Evaluation of BER-LC-CTS-NPs in a Diabetic Animal Model

#### 2.4.1. Ethical Considerations and Animal Care

Following acceptance from the Animal Ethics Committee at Beni-Suef University, 24 Wistar rats weighing 170–210 g were employed. The animals were housed in propylene cages after acclimatization and kept under controlled temperature (25 ± 2 °C) with light and dark cycles (12:12 h). The rats were fed standard animal feed and had unlimited access to water. 

#### 2.4.2. Induction of Diabetes Mellitus (DM) 

The animals were given a 10% fructose solution ad libitum for 15 days prior to DM induction [34]. After that, the animals returned for unlimited water. Before the administration of STZ, all animals were subjected to a day of fasting, a time of low movement and stress. Each animal was given streptozocin (50 mg/kg) dissolved in citrate buffer with pH 4.4 (20 mg/mL) intraperitoneally. To ensure optimal streptozocin concentration to cause DM, the volume of injection was calculated based on the body weight of each animal. To check for DM induction 72 h after streptozocin injection, blood samples were obtained from the animal’s tail veins and glucose levels were assessed via a commercial glucometer (GlucoDr®, Gyeonggi-do, Korea). The animals were categorized as diabetic if their blood glucose level exceeded 200 mg/dL [35,36,37] and the presence of the following symptoms confirmed the condition: polyuria, polyphagia, and polydipsia [34]. Every three days, the animal’s glycemia was checked to ensure that blood glucose levels remained stable.

#### 2.4.3. Wound Creation 

The wound was created using the procedure described by Correa et al. [38]. Ten days following DM confirmation, the rats were anesthetized with an intraperitoneal xylazine (5 mg/kg) and ketamine (90 mg/kg). The dorsal area between the animal shoulder blades was shaved and disinfected with 70% ethanol. A 10 mm diameter sterile biopsy punch was used to create circular wounds. Following that, the skin and subcutaneous tissue were removed, leaving the muscles exposed.

#### 2.4.4. Wound Treatment 

Diabetic rats were split into four major groups, each with six animals, based on the treatment they received: (A) animals served as untreated control negative (CG), (B) animals treated with lecithin–chitosan nanoparticles without BER (LC-CTS-NPs), (C) animals treated with free BER, (D) animals treated with BER loaded lecithin–chitosan nanoparticles (BER-LC-CTS-NPs). The application of the treatment on the wounds commenced from the day at which the wound was created (day 0) and continued on a daily basis for fourteen consecutive days at the same time (12 pm). The wounds were cleansed with sterile gauze soaked with saline solution before placing the treatments (1 gm) above the wound. Throughout the experiment, the wounds were left uncovered.

### 2.5. Treatment Evaluation (Macroscopic Analyses)

In the excised model, the wound area was determined by tracing the wound using a transparent millimeter scale, and digital images were taken on days 0, 3, 6, 9, 12, and 14 for all groups. Wound area measurements were used as an estimation parameter [37,39]. The wound closure percentage was estimated by taking the original size of the wound and using the following equation on every fixed day before the animal’s wound healed completely

$$\mathrm{Wound}\;\mathrm{closure}\;\mathrm{rate}\;\left(\%\right)=\frac{\left(W_{0}-W_{t}\right)}{W_{0}}\times 100$$

where W_0_ = initial wound area, and W_t_ = reported wound area. 

### 2.6. Histopathological Evaluation (Microscopic Analyses) 

Skin wound samples with their edges were dissected from the diabetic rats in all studied groups, rapidly fixed in 10% formalin for 48 h, then exposed to routine paraffin histological techniques and following staining according to Suvarna et al. [40]. The different stages of healing were analyzed and evaluated on the 3rd, 7th, and 14th days post wound creation by:

Measuring of wound gap length and granulation tissue thickness in sections stained with H&E ×40.

Estimation of inflammatory cell infiltration, fibroblast proliferation, and blood vessel counts in sections stained with H&E ×400 [38].

Quantification of total collagen fibers area percentage in sections stained with Crossman’s trichrome and Picrosirius red stains under the light microscope (×100). 

Quantification of mature collagen fibers area percentage in sections stained with Picrosirius red stain ×200 under the polarized microscope [38].

LEICA (DFC290 HD system digital camera, Switzerland) was used to examine and photograph all stained sections. Also, the Picrosirius red-stained sections were captured by Olympus BX-53P polarizing microscope [41]. The analysis was performed by Image-J program 1.52a freeware on 3 fields × 3 slides in each group.

## 3. Statistical Analysis 

Each test was repeated three times, and the findings were presented as mean ± standard deviation (SD). To assess statistical differences between classes, one-way ANOVA with Tukey post hoc test was used where applicable. For the microscopic assessment, SPSS software (version 22, SPSS Inc., Chicago, IL, USA) was used for the evaluation. To differentiate between groups, a mixed ANOVA followed by a Bonferroni post-test was utilized. *p*-value < 0.05 in the research was regarded significant

## 4. Results and Discussion

### 4.1. Design of Experiments and Optimization

The composition and characteristics of the formulations of BER-LC-CTS-NPs (F1-F17) are shown in Table 2. The relations between the formulation variables: LC amount (X_1_), CTS amount (X_2_), and IPM concentration (X_3_), and the response variables (particle size (PS: Y1), zeta potential (ZP: Y2), and entrapment efficiency (EE %:Y3)) were optimally represented according to the equations

PS = 178.382 + 4.1125 × X_1_ + 43.475 × X_2_ + 13.5625 × X_3_

Ln (ZP) = 3.36885 − 0.0713389 × X_1_ + 0.278543 × X_2_ + 0.00321433 × X_3_ − 0.10896 × X_2_^2^

EE = 72.5489 + 0.87625 × X_1_ + 3.995 × X_2_ + 16.9863 × X_3_ − 1.1175 × X_2_ × X_3_ − 7.47264 × X_3_^2^

**Figure 1:**
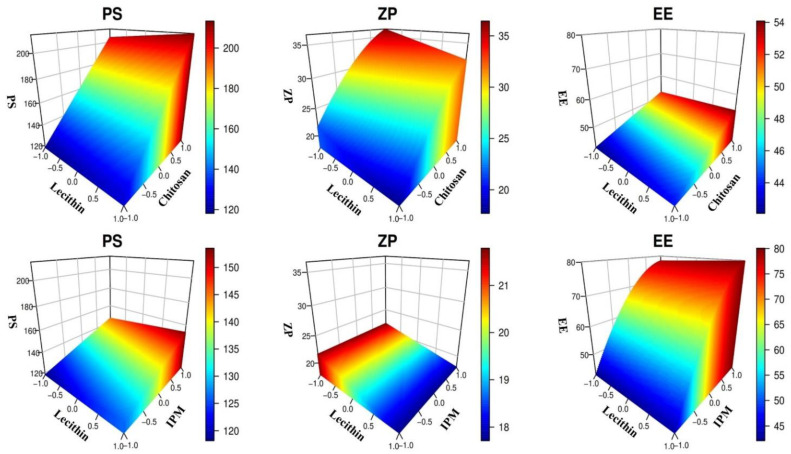
3D response surface plots for the influences of LC amount, CTS amount, and IPM concentrations on BER-LC-CTS-NPs PS, ZP, and EE%.

As presented in Supplementary Table S1 in the supplementary file, the models adequately described the dispersion of measured data, as demonstrated by the negligible lack of fit error (*p* ≥ 0.1). Visual analysis of the diagnostic model plots shows that the models sufficiently fit the responses without evidence of residual error patterns or non-normal distribution (Supplementary Figures S1–S3). The equations were explored graphically in Figure 1 to map the response area produced from the interplay of the formulation factors. 

The left column of Figure 1 indicates that increasing CTS or IPM level produces large particles, while the effect of LC on PS is insignificant. When CTS amount increased from 10 to 40 mg, the PS size increased from 140 nm to >200 nm in all levels of LC amount (Figure 1, left upper panel). These outcomes align with the results obtained by Alkholief, who showed that increasing CTS level results in larger particles [42]. Moreover, increasing the amount of IPM from 1% to 3% resulted in increases in the PS from 160 nm to more than 180 nm in all levels of LC amount (Figure 1, left lower panel). This behavior is because IPM contributes to the lipidic core of the nanoparticles [43]. 

The middle upper panel of Figure 1 indicates that raising the amount of CTS from 10 to 40 mg increased ZP from +21.5 to >+35 mV. This increase in charge is attributed to an increase in amino acid concentration, which gives BER-LC-CTS-NPs a further positive charge [42]. Additionally, increasing LC amount from 100 to 200 mg resulted in a decrease in the ZP from +21 to +18 mV (Figure 1, the middle upper panel). This drop in the surface charge is due to an increase in the phosphate negative groups (PO^3−^) of LC [44]. On the other hand, the IPM had a negligible effect on ZP (Figure 1, the middle lower panel).

The right upper panel of Figure 1 indicates that increasing the amount of LC from 100 to 200 mg leads to a slight increase in EE from 67% to 69%. This is in line with the outcomes presented by Khalil et al. [45]. Besides, increasing the amount of CTS from 10 to 40 mg increased the EE from 67% to >75% (Figure 1, right upper panel). These results are in accordance with that of Alkholief, who reported that increasing CTS level led to a slight increase in EE [42]. Interestingly, increasing IPM level from 1% to 3%, led to a remarkable increase in EE from 47% to more than 80% (Figure 1, right lower panel). Due to the lipophilic nature of BER, it is stationed within the lipid core of LC-CTS-NPs, whose lipid content is altered by the existence of IPM. Accordingly, the encapsulation efficiency is prone to enhancement by the inclusion of IPM in the formulation of nanoparticles [46].

**Figure 2:**
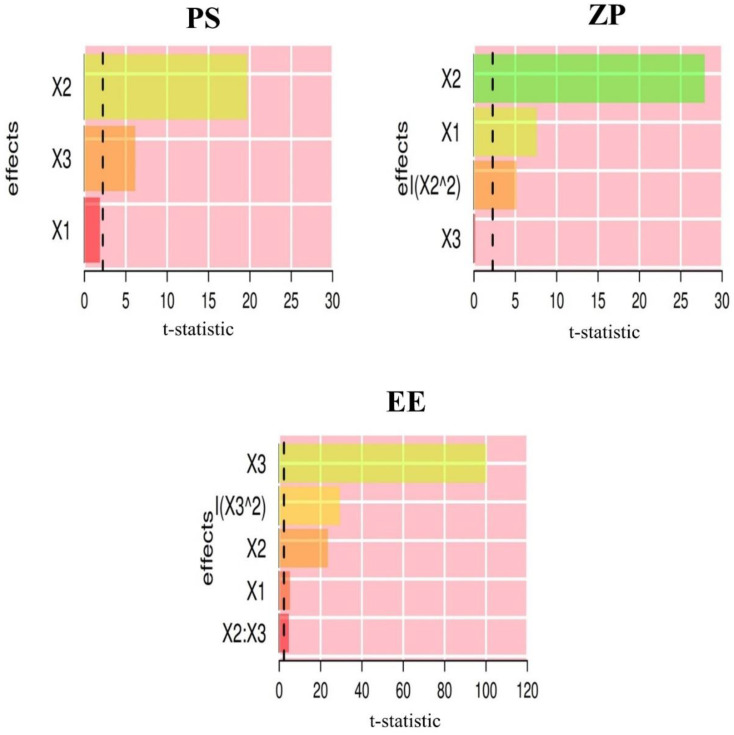
Pareto chart depicts the standardized impacts of LC amount (X_1_), CTS amount (X_2_), and IPM concentration (X_3_). The bars represent the *t*-statistics (which were calculated as parameter estimate/estimate standard error). The critical *t*-value is represented by the dashed lines (the 95th percentile of a two-tail t-distribution with error degrees of freedom).

The Pareto chart (Figure 2) shows the standardized relative effects of the formulation factors on size, surface charge, and entrapment. Particle size and surface charge were more influenced by the CTS than the LC and IPM levels. However, IPM level was the most influential for EE. Table 3 lists the constituents of the optimized BER-LC-CTS-NPs formulation. Using our models, the features of the optimal LC-CTS-NPs were accurately predicted as all responses showed very low prediction error (<8%, Table 3).

### 4.2. Characterization of the Optimized BER-LC-CTS-NPs 

#### 4.2.1. In Vitro Release Study of BER 

**Figure 3:**
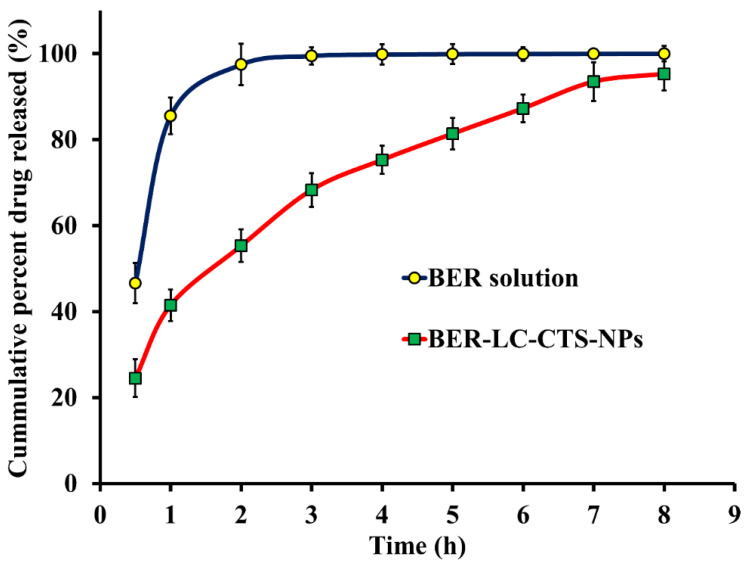
In vitro release profile study of BER from optimized BER-LC-CTS-NPs and BER solution through dialysis membranes.

The BER release profile from optimized BER-LC-CTS-NPs and the BER solution is shown in Figure 3. The optimized nanoparticles showed a sustained release behavior, with a burst release period of 0 to 3 h and a steady release period of 3 to 8 h. The first burst may be attributed to the hydrophilization of the surface of the nanoparticles caused by formation in a polar solvent such as ethanol [47]. The subsequent sustained release behavior is due to swelling of the hydrophilic molecules of CTS in an aqueous environment [48]. The ability of the nanoparticles to sustain the delivery of BER to the wound site can be expected to prolong the drug’s wound healing effect. Moreover, the bioadhesive properties of CTS [23] can increase the residence time of the nanoparticles on the wound site, thus avoiding rapid clearance of the drug and increasing the local concentration of the drug at the site of administration.

#### 4.2.2. The Morphology and pH of the Optimized BER-LC-CTS-NPs

**Figure 4:**
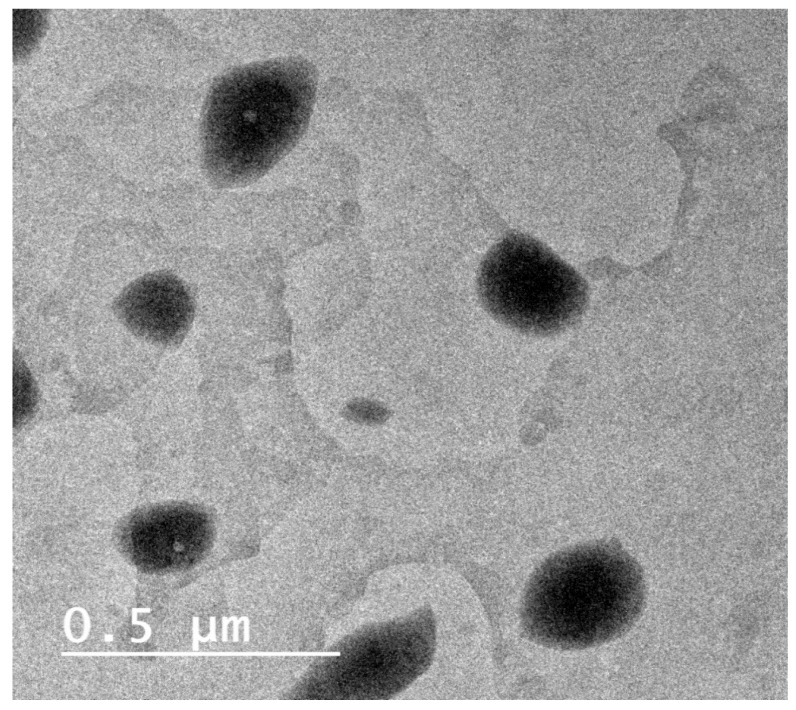
Transmission electron microscope image of BER-LC-CTS-NPs optimal formula.

Spherical particles were seen in TEM images of the optimized BER-LC-CTS-NPs (Figure 4), and the particles were distinguished by a contrast corona, representing CTS, circling a lipid core, composed of LC and IPM, in which the compound was fairly dissolved. As noted by both Zetasizer and TEM, a good correlation was obtained in the PS. The pH value of the optimized BER-LC-CTS-NPs was 5.43. 

#### 4.2.3. Physical Stability of the Optimized BER-LC-CTS-NPs

**Figure 5:**
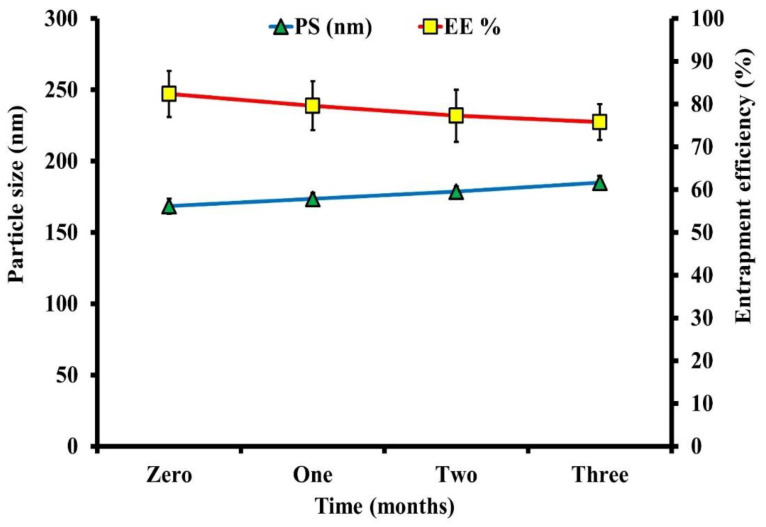
The effect of 3 months of storage at 4 °C on the PS and EE% of the optimized BER-LC-CTS-NPs formulation.

As illustrated in Figure 5, the optimized BER-LC-CTS-NPs showed minimal changes in PS (size increased from 168.4 ± 5.31 nm to 184.9 ± 4.83 nm) and EE% (entrapment decreased from 82.4 ± 4.9% to 75.8 ± 3.9) during the three months. These differences were deemed to be insignificant (*p* > 0.05 on one-way ANOVA). The stability of optimized BER-LC-CTS-NPs may be attributed to their high surface charge (+33.1 mV). Formulations are generally considered stable if their surface charge was more than +30 mV or less than-30 mV [49,50].

### 4.3. In Vivo Investigations in the DM Rat Model

#### Macroscopic Analyses

**Figure 6:**
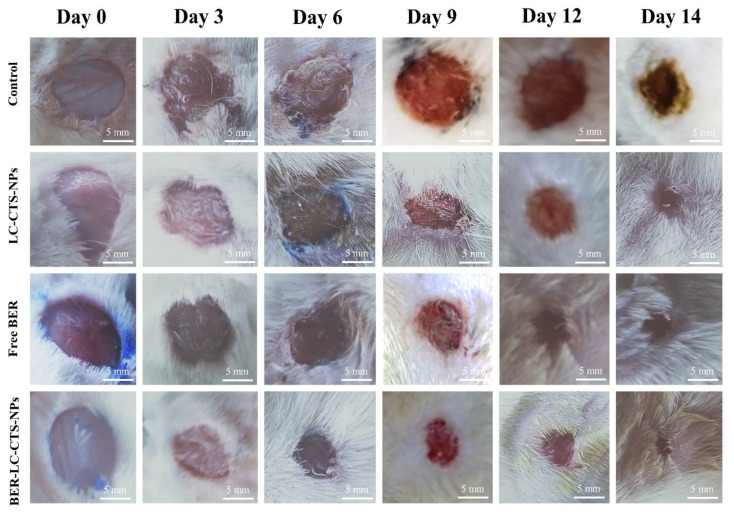
Photograph images of wound repair on the 0th, 3rd, 6th, 9th, 12th, and 14th day in the diabetic rats of the untreated (Control), lecithin–chitosan nanoparticles without berberine (LC-CTS-NPs), free berberine (Free BER), and lecithin–chitosan nanoparticles containing berberine (BER-LC-CTS-NPs) groups.

**Figure 7:**
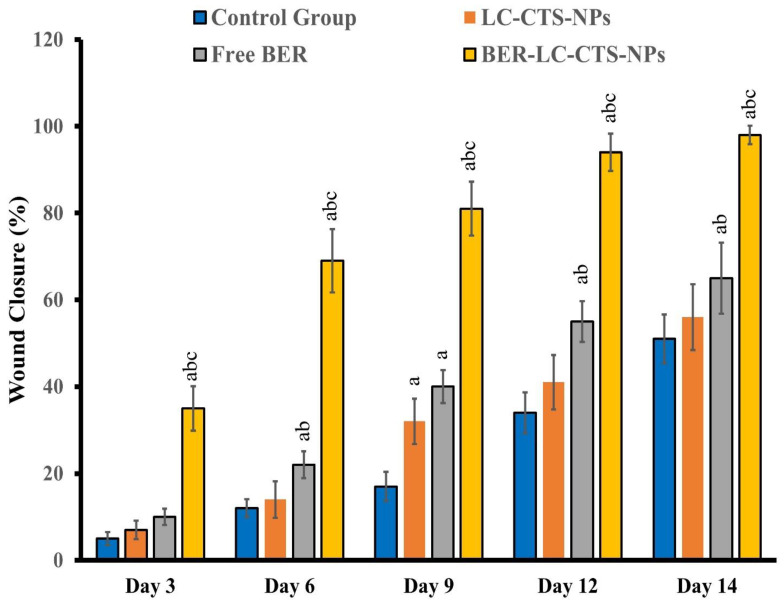
Percent wound closure rate of wound area in treated animals on the 3rd, 6th, 9th, 12th, and 14th day after wound creation in the diabetic rats of the untreated (Control), lecithin–chitosan nanoparticles without berberine (LC-CTS-NPs), free berberine (Free BER), and lecithin–chitosan nanoparticles containing berberine (BER-LC-CTS-NPs) groups. ^a^ illustrates a significant variation when compared to the Control group. ^b^ illustrates a significant variation when compared to LC-CTS-NPs group. ^c^ illustrates a significant variation when compared to Free BER group.

Figure 6 and Figure 7 demonstrate the macroscopic characteristics of the wounds and the progression of the wound region, with improved wound-closing in animals treated with free BER, LC-CTS-NPs, and BER-LC-CTS-NPs. Figure 6 depicts the photograph of the most representative rat belonging to each treatment group. The wounds of all groups, except for the BER-LC-CTS-NPs group, remained open until the ninth day. The wound of the BER-LC-CTS-NPs treated group was nearly closed by the ninth day. When compared to the control, free BER, and LC-CTS-NPs groups on day14, the rats treated with BER-LC-CTS-NPs demonstrated a significant difference in wound area reduction (Figure 7).

Several researches have been performed recently to improve the impact of BER on wound healing. For instance, Zhang et al. synthesized BER nanohydrogel from polyvinyl alcohol and sodium alginate [14]. According to the molecular mechanism and animal studies, BER nanohydrogel activates Sirt1, which promotes wound healing by decreasing inflammation and boosting angiogenesis. Additionally, Samadian et al. recently developed an electrospun Cellulose Acetate/Gelatin mat loaded with BER for the treatment of diabetic foot ulcers [51]. Experimentation on streptozocin-induced diabetic rats showed that the CA/Gel bandage containing BER promoted wound healing. Furthermore, Amato et al. developed a nanogel platform composed of hyaluronan, poly-L-lysine, and BER and evaluated it in vitro [52]. The results showed that a nanogel loaded with BER could completely close the gap between fibroblasts after 42 h. Interestingly, our developed lecithin–chitosan-based nanoparticulate system loaded with BER was able to speed up the repair process in streptozocin-induced diabetic rats and bring the wound gap to almost complete closure within 14 days. The cohabitation of the two components, CTS and BER, has a beneficial effect on the wound healing process, as evidenced by the greater wound closure rate in group (D), the group that received BER-LC-CTS-NPs. Both components have been shown to aid in wound healing [14,20,23,51,52,53,54,55].

### 4.4. Microscopic Analyses

#### 4.4.1. Evaluation of Healing Process in the Skin Wound Using H&E Stain

**Figure 8:**
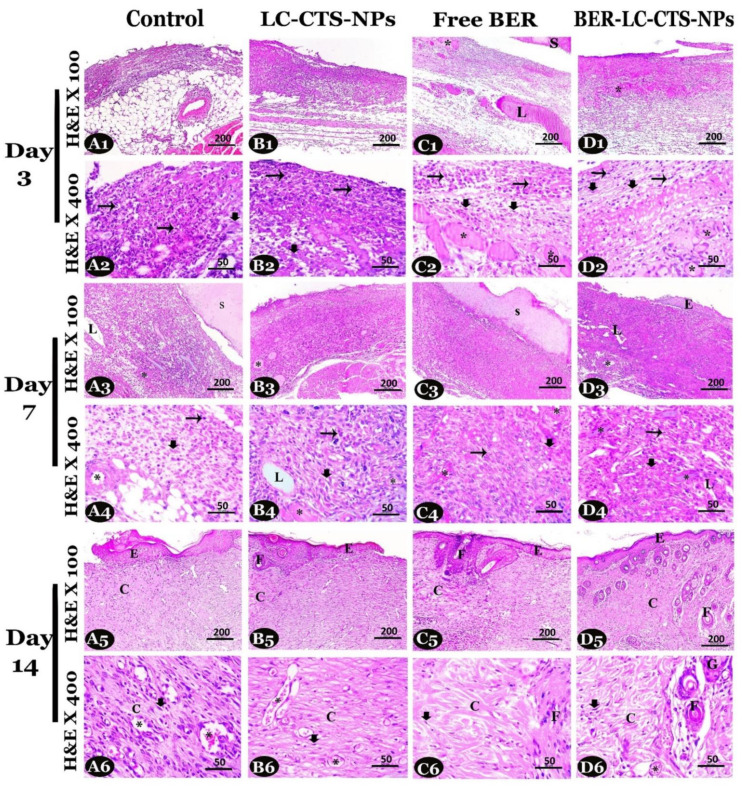
Photomicrographs of H&E stained skin sections (1st, 3rd and 5th rows ×100, and 2nd, 4th and 6th rows ×400) on days 3, 7 and 14 post wound creation in the diabetic rats of the Control (**A1**–**A6**), LC-CTS-NPs (**B1**–**B6**), Free BER (**C1**–**C6**) and BER-LC-CTS-NPs (**D1**–**D6**) groups. Note: inflammatory cells (long arrows), fibroblast proliferation (thick arrows), blood vessels (*), lymph vessels (L), scar (S), newly formed epidermis (E), collagen fibers (C), hair follicles (F), and sebaceous gland (G).

In our study, we have demonstrated the role of free BER and BER-LC-CTS-NPs in wound healing in diabetic rats. Representative H&E stained skin sections ×400 presented in Figure 8 were used for general evaluation of regenerated tissue in all studied groups. Wound sections stained with H&E ×400 were used for evaluation of wound gap length (Table 4a) and granulation tissue thickness (Table 4b) in all studied groups. Due to the presence of special subcutaneous muscle in rats, the contraction of wound edges is rabid and easy [56]. It was noted that BER-LC-CTS-NPs treatment further accelerated wound healing in diabetic rats. We observed that, on days 7 and 14, the reduction of wound gap in Free BER and BER-LC-CTS-NPs groups was greater than the other groups. The Free BER and BER-LC-CTS-NPs groups revealed more complete re-epithelialization and gap closure on day 14 compared to the other groups (Table 4a). The newly formed granulation tissue thickness was significantly increased on days 3 and 14 with Free BER and BER-LC-CTS-NPs treatments compared to the other treatments (Figure 8 and Table 4b). BER-LC-CTS-NPs treatment showed the formation of skin appendages on day 14. Therefore, the regenerated tissue appeared thicker in the BER-LC-CTS-NPs group.

On all days, a higher intensity of inflammatory cells could be spotted in the control group compared to the BER-LC-CTS-NPs group, which revealed the least intensity (Table 4c). On days 3 and 7, the LC-CTS-NPs, Free BER, and BER-LC-CTS-NPs treatments significantly increased fibroblast proliferation compared to the control. Thus, treatment with BER-LC-CTS-NPs shortened the inflammatory phase, reduced the inflammatory signs, and activated the early proliferative phase (Table 4c,d). On day 14, fibroblast was significantly reduced in the BER-LC-CTS-NPs group compared to the other groups (Table 4d). We observed a spike in the number of blood vessels in the Free BER and BER-LC-CTS-NPs groups in relation to the control group on days 3 and 7. On day14, the largest number of vessels appeared in the control and LC-CTS-NPs groups (Table 4e), owing to the fact that re-epithelialization and tissue regeneration had not yet been achieved.

#### 4.4.2. Identification and Evaluation of Collagen Fibers Area Percentage in the Skin Wound

**Figure 9:**
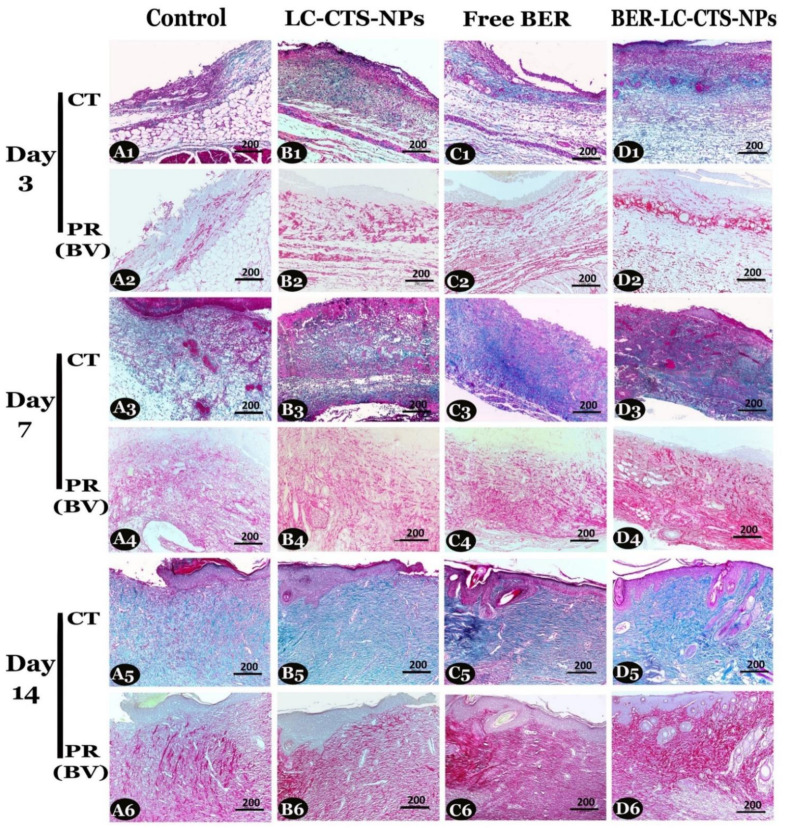
Representative skin sections ×100 used for collagen quantification, on days 3, 7, and 14 post wound creation in the diabetic rats of the Control (**A1**–**A6**), LC-CTS-NPs (**B1**–**B6**), Free BER (**C1**–**C6**), and BER-LC-CTS-NPs (**D1**–**D6**) groups. Collagen fibers are stained green with Crossman’s trichrome (CT) (1st, 3rd, and 5th rows) and red with Picrosirius red stain (bright-field view (PR (BV)) (2nd, 4th, and 6th rows). Note: in all days, collagen fibers were the highest in the Free BER and BER-LC-CTS-NPs groups. On day 14, fibers were organized as short bundles in wound borders of all groups but appeared also in the wound center in the Free BER and BER-LC-CTS-NPs groups only.

By using Crossman’s trichrome (CT) and Picrosirius red (bright-field view) (PR (BV)) stains, representative wound sections ×100, presented in Figure 9, were used for total collagen fiber identification and quantification. The evaluation of total collagen area percentage using CT stain (green fibers) is shown in Table 5a, and PR (BV) stain (red fibers) is shown in Table 5b) On all days, a significant difference in collagen quantity was spotted between all groups (*p* < 0.05). Both the Free BER and BER-LC-CTS-NPs groups showed the highest amount of collagen compared to the control and LC-CTS-NPs groups. On day 14, the organization of fibers as short bundles in both the wound borders and the center were clearly observed in the Free BER and BER-LC-CTS-NPs groups (Figure 9(C5,6,D5,6). It seems that the nanoparticles containing BER enhanced the stimulation of blood vessels, fibroblast proliferation, and collagen deposition leading to early closure of the wound. 

#### 4.4.3. By Using Picrosirius Red Stain (Polarized-Field View) (PR (PV))

**Figure 10:**
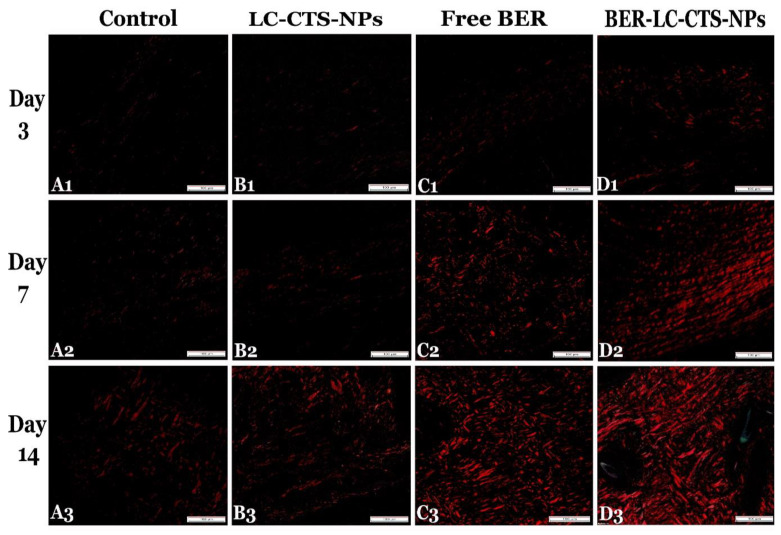
Representative skin sections on days 3, 7, and 14 post wound creation, stained with Picrosirius red stain (Polarized-field view ×200) for mature collagen fibers quantification in the diabetic rats of the Control (**A1**–**A3**), LC-CTS-NPs (**B1**–**B3**), Free BER (**C1**–**C3**), and BER-LC-CTS-NPs (**D1**–**D3**) groups. Note: in all days, the largest amount of mature fibers was formed in the BER-LC-CTS-NPs treated group (**D1**–**D3**) and was organized on day 14 as thick short bundles running in the same direction (**D3**).

Representative wound sections ×200, presented in Figure 10, were used for evaluation of the area percentage of mature collagen (type I (red polarized fibers)) using PR (PV) stain under the polarizing microscope. The deposition and organization of the fibers were significantly different between all groups, *p* ≤ 0.05 (Table 5c). On all days, the Free BER and BER-LC-CTS-NPs groups revealed a significantly large amount of mature collagen fibers. The highest percentage of collagen on day 14 was present in the BER-LC-CTS-NPs group and appeared as short bundles running in the same direction compared to the other groups (Figure 10(A3,B3,C3,D3)).

## 5. Conclusions

This study is the first to introduce lecithin–-chitosan nanoparticles loaded with berberine as a novel agent in healing wounds induced in diabetic rats. The optimized nanoparticles showed a mean size of 168.4 nm, surface charge of 33.1 mV, entrapment of 82.3%, and good stability as well as sustained release behavior to control inflammation. The in vivo study proved that combining chitosan and berberine in the nanoparticles produces a synergistic effect when it comes to wound healing. The optimized nanoparticulate system works by reducing inflammation, inducing blood vessels and fibroblast proliferation, and promoting mature collagen fibers deposition. Based on the experimental results, lecithin–chitosan nanoparticles loaded with berberine have evolved as a promising strategy for accelerating the wound healing process in diabetic patients. However, the clinical merits of the developed system need to be investigated in diabetic patients.

## Figures and Tables

**Table 1:** The coded values of various variables used for formulation of BER-LC-CTS-NPs in the Box–Behnken design.

Factor	Levels		
	−1	0	1
X_1_: Lecithin amount (mg)	100	150	200
X_2_: CTS amount (mg)	10	25	40
X_3_: IPM concentration in ethanolic solution (% *w*/*v*)	1	2	3
Dependent variables	Applied constrains		
Y1:particle size (nm)	Minimize		
Y2: zeta potential (mV)	Maximize		
Y3: entrapment efficiency (%)	Maximize		

**Table 2:** BER-LC-CTS-NPs experimental runs, independent variables, and response variables according to the Box–Behnken design.

No.	X_1_ Lecithin Amount (mg)	X_2_ CTS Amount (mg)	X_3_ IPM (% *w*/*v*)	Y_1_ PS (nm)	Y_2_ ZP (mV)	Y_3_ EE (%)
1	150	10	1	116.4 ± 5.33	19.1 ± 0.3	43.32 ± 3.4
2	150	25	2	175.0 ± 10.4	28.3 ± 1.3	72.79 ± 5.2
3	100	10	2	125.4 ± 7.95	21.7 ± 0.9	67.21 ± 4.2
4	200	25	3	196.4 ± 11.3	27.9 ± 1.7	82.37 ± 6.2
5	150	40	3	242.1 ± 12.3	34.8 ± 2.3	84.97 ± 4.9
6	200	40	2	219.2 ± 7.14	31.2 ± 1.9	77.36 ± 2.7
7	150	25	2	178.6 ± 8.26	29.6 ± 2.4	73.44 ± 4.3
8	100	25	1	171.3 ± 6.41	31.5 ± 2.8	46.74 ± 3.9
9	150	40	1	211.1 ± 9.79	35.1 ± 3.2	53.37 ± 4.7
10	200	25	1	173.8 ± 10.6	27.1 ± 2.4	48.93 ± 2.5
11	200	10	2	141.9 ± 5.23	18.5 ± 0.6	69.53 ± 3.6
12	150	10	3	157.7 ± 4.62	19.7 ± 1.2	79.39 ± 5.1
13	100	40	2	216.8 ± 13.1	36.8 ± 2.7	75.71 ± 4.5
14	100	25	3	184.9 ± 5.84	30.7 ± 2.5	81.52 ± 3.7
15	150	25	2	180.0 ± 4.92	27.8 ± 1.6	74.37 ± 6.3
16	150	25	2	168.4 ± 3.47	28.8 ± 2.3	71.73 ± 3.1
17	150	25	2	173.5 ± 6.28	30.0 ± 2.9	70.95 ± 4.6

**Table 3:** Composition, experimental estimated value, and model expected value of the optimized BER-LC-CTS-NPs formulation.

Independent Factors	Optimal Value	Response Variables	Estimated Value	Model Expected Value	Prediction Error (%) *
X_1_: Lecithin amount (mg)	100	PS (nm)	168.4	179.1	6.4
X_2_: CTS amount (mg)	23.5	ZP (mV)	33.1	30.5	7.9
X_3_: IPM concentration in ethanolic solution (% *w/v*)	2.6	EE%	82.3	79.2	3.8

* Calculated as (estimated−expected)/estimated × 100.

**Table 4:** Microscopic evaluation of wound healing of the skin in the diabetic rats of the Control, LC-CTS-NPs, Free BER, and BER-LC-CTS-NPs treated groups using sections stained with H&E ×40 (**a**,**b**) and sections stained with H&E ×400 (**c**–**e**). Data were expressed as mean ± SD (*n* = 3).

**Day**	**(a) Wound Gap Length (µm)**			
	**Control**	**LC-CTS-NPs**	**Free BER**	**BER-LC-CTS-NPs**
Day 3	17,297 ± 1025	15,191 ± 455 ^a^	13,933 ± 206 ^a^	13,130 ± 271 ^ab^
Day 7	14,312 ± 655	10,955 ± 934 ^a^	9160 ± 711 ^a^	7406 ± 400 ^ab^
Day 14	4697 ± 994	1623 ± 514 ^a^	106 ± 32 ^ab^	33 ± 9 ^ab^
**Day**	**(b) Granulation Tissue Thickness (Height) (µm)**			
	**Control**	**LC-CTS-NPs**	**Free BER**	**BER-LC-CTS-NPs**
Day 3	1977 ± 254	2220 ± 202	4284 ± 275 ^ab^	4916 ± 124 ^abc^
Day 7	3513 ± 103	4172 ± 189 ^a^	5030 ± 27 ^ab^	5222 ± 69 ^ab^
Day 14	4547 ± 479	4908 ± 199	6159 ± 120 ^ab^	7422 ± 200 ^abc^
**Day**	**(c) Inflammatory Cell Infiltration**			
	**Control**	**LC-CTS-NPs**	**Free BER**	**BER-LC-CTS-NPs**
Day 3	140.3 ± 7.2	128.0 ± 8.7	127.3 ± 8.6	122.3 ± 4.0
Day 7	98.7 ± 4.0	89.7 ± 4.7	80.0 ± 6.1 ^a^	70.0 ± 3.0 ^ab^
Day 14	33.3 ± 4.2	27.3 ± 2.5	25.0 ± 2.6	25.7 ± 4.1
**Day**	**(d) Fibroblast Proliferation**			
	**Control**	**LC-CTS-NPs**	**Free BER**	**BER-LC-CTS-NPs**
Day 3	8.0 ± 2.0	12.7 ± 1.5 ^a^	22.0 ± 3.0 ^ab^	25.0 ± 2.6 ^ab^
Day 7	43.0 ± 3.0	55.3 ± 2.5 ^a^	51.3 ± 3.1 ^a^	56.7 ± 1.5 ^a^
Day 14	75.3 ± 4.5	73.3 ± 1.5	67.0 ± 4.6	64.7 ± 1.3 ^a^
**Day**	**(e) Blood Vessels Count**			
	**Control**	**LC-CTS-NPs**	**Free BER**	**BER-LC-CTS-NPs**
Day 3	3.0 ± 2.0	8.0 ± 1.0	11.0 ± 2.0 ^a^	13.0 ± 2.6 ^a^
Day 7	8.0 ± 2.0	11.0 ± 1.7	15.0 ± 1.0 ^a^	16.3 ± 1.5 ^ab^
Day 14	25.3 ± 1.5	22.0 ± 1.0 ^a^	12.0 ± 1.0 ^ab^	12.3 ± 0.6 ^ab^

^a^ illustrates a significant variation when compared to the Control group. ^b^ illustrates a significant variation when compared to LC-CTS-NPs group. ^c^ illustrates a significant variation when compared to Free BER group.

**Table 5:** Microscopic evaluation for collagen fiber quantification during the healing process in the diabetic rats of all studied groups; by measuring the area percentage of total collagen fibers using sections stained with Crossman’s trichrome stain ×100 (**a**) and Picrosirius red stain (bright-field view) ×100 (**b**), and measuring the area percentage of mature collagen fibers using sections stained with Picrosirius red stain (Polarized-field view) ×200 (**c**). Data were expressed as mean ± SD (*n* = 3).

**Day**	**(a) Total Collagen Area Percentage (Crossman’s Trichrome Stain)**			
	**Control**	**LC-CTS-NPs**	**Free BER**	**BER-LC-CTS-NPs**
Day 3	3.6 ± 0.3	4.2 ± 0.1	4.6 ± 0.3 ^a^	6.9 ± 0.4 ^abc^
Day 7	7.2 ± 0.5	8.0 ± 0.6	10.1 ± 0.2 ^ab^	15.2 ± 0.3 ^abc^
Day 14	13.4 ± 0.2	15.3 ± 0.2 ^a^	20.2 ± 0.4 ^ab^	28.6 ± 0.6 ^abc^
**Day**	**(b) Total Collagen Area Percentage (Picrosirius Red Stain)** **(Bright View)**			
	**Control**	**LC-CTS-NPs**	**Free BER**	**BER-LC-CTS-NPs**
Day 3	3.78 ± 0.21	5.84 ± 0.26 ^a^	7.92 ± 0.20 ^ab^	9.47 ± 0.22 ^abc^
Day 7	8.05 ± 0.18	9.96 ± 0.19 ^a^	11.98 ± 0.21 ^ab^	17.36 ± 0.21 ^abc^
Day 14	14.98 ± 0.24	18.09 ± 0.30 ^a^	22.74 ± 0.66 ^ab^	29.38 ± 0.76 ^abc^
**Day**	**(c) Mature Collagen Fibers Area Percentage (Picrosirius Red Stain)** **(Polarized-Field View)**			
	**Control**	**LC-CTS-NPs**	**Free BER**	**BER-LC-CTS-NPs**
Day 3	3.0 ± 0.11	3.1 ± 0.15	4.4 ± 0.60 ^ab^	5.0 ± 0.15 ^ab^
Day 7	6.2 ± 0.09	6.7 ± 0.20	8.7 ± 0.21 ^ab^	12.6 ± 0.35 ^abc^
Day 14	11.8 ± 0.20	12.6 ± 0.35 ^a^	17.0 ± 0.18 ^ab^	20.9 ± 0.30 ^abc^

^a^ illustrates a significant variation when compared to the Control group. ^b^ illustrates a significant variation when compared to LC-CTS-NPs group. ^c^ illustrates a significant variation when compared to Free BER group.

## Data Availability

Not applicable.

## Supplementary Materials

The following are available online at https://www.mdpi.com/article/10.3390/pharmaceutics13081197/s1, Figure S1: Plot of observed versus model predicted responses, Figure S2: Normal quantile-quantile plots of residual errors, Figure S3: Plot of residual error versus model predicted responses, Table S1: Analysis of variance (ANOVA) of the final models for measured responses.
