# Supplementary material for: Berberine Encapsulated Lecithin–Chitosan Nanoparticles as Innovative Wound Healing Agent in Type II Diabetes
Source: Pharmaceutics. 2021 Aug 4;13(8):1197. doi: 10.3390/pharmaceutics13081197 (PMC8401853; doi:10.3390/pharmaceutics13081197)
Supplement: Supplementary file 1 [file pharmaceutics-13-01197-s001.zip › pharmaceutics-1304230-supplementary.pdf]

pharmaceutics

MDPI

Supplementary Materials: Berberine Encapsulated Lecithin–Chitosan Nanoparticles as Innovative Wound Healing Agent in Type II Diabetes

Dibya Sundar Panda, Hussein M. Eid, Mohammed H. Elkomy, Ahmed Khames, Randa M. Hassan, Fatma I. Abo El-Ela and Heba A. Yassin

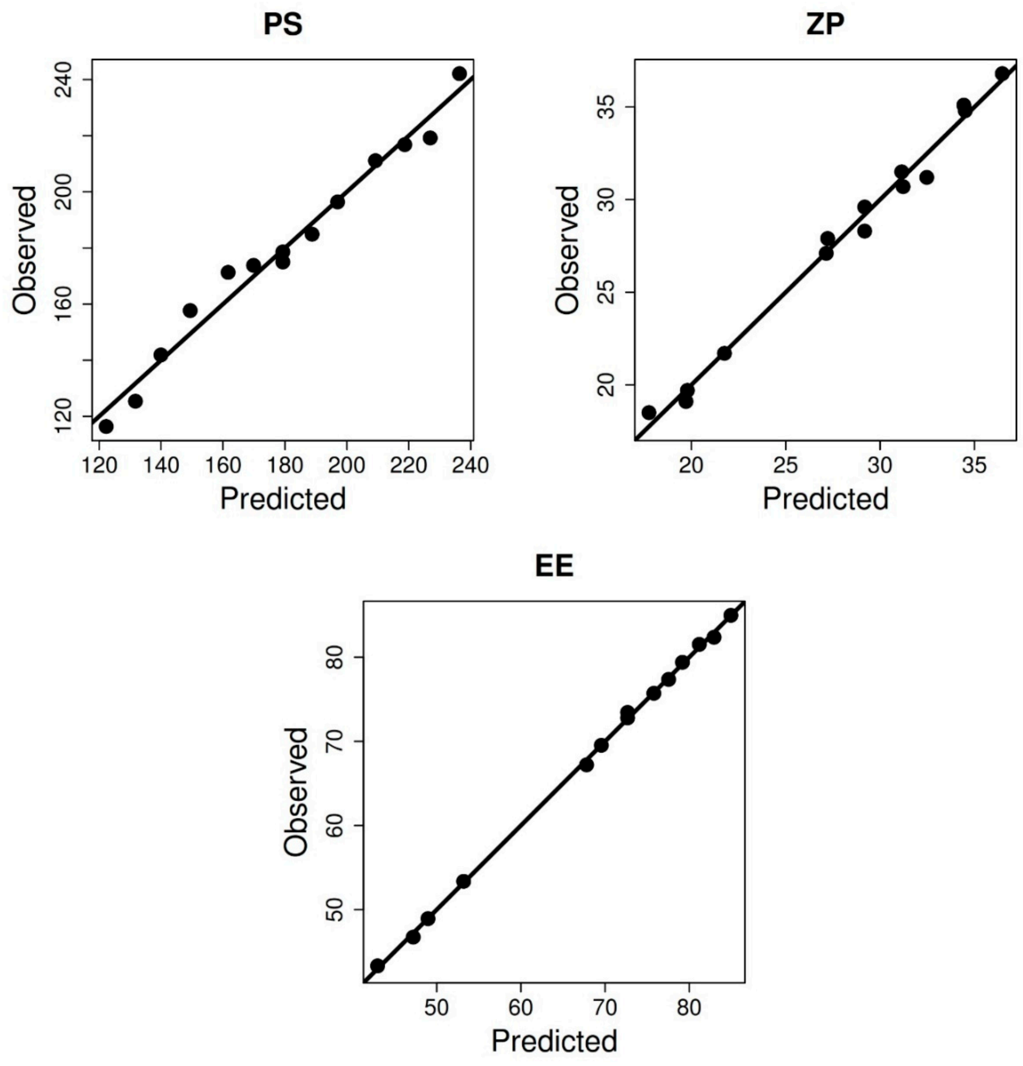

Figure S1. Plot of observed versus model predicted responses.

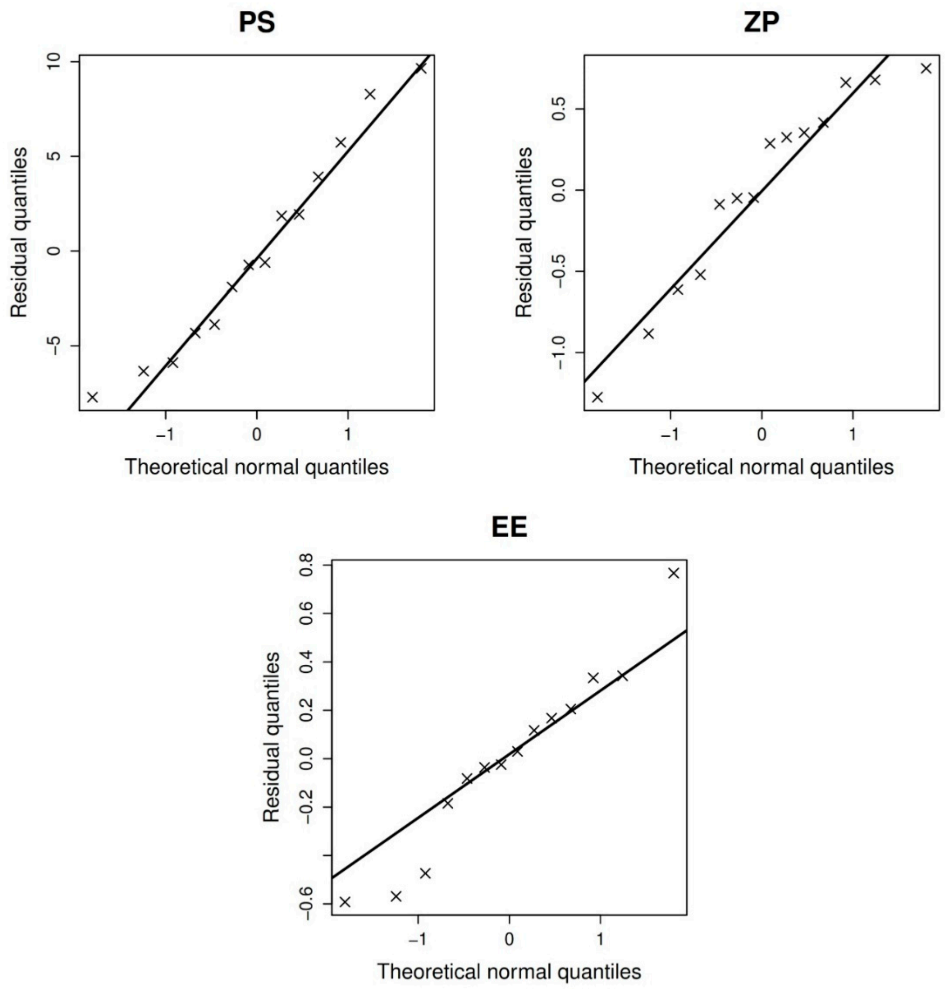

Figure S2. Normal quantile-quantile plots of residual errors.

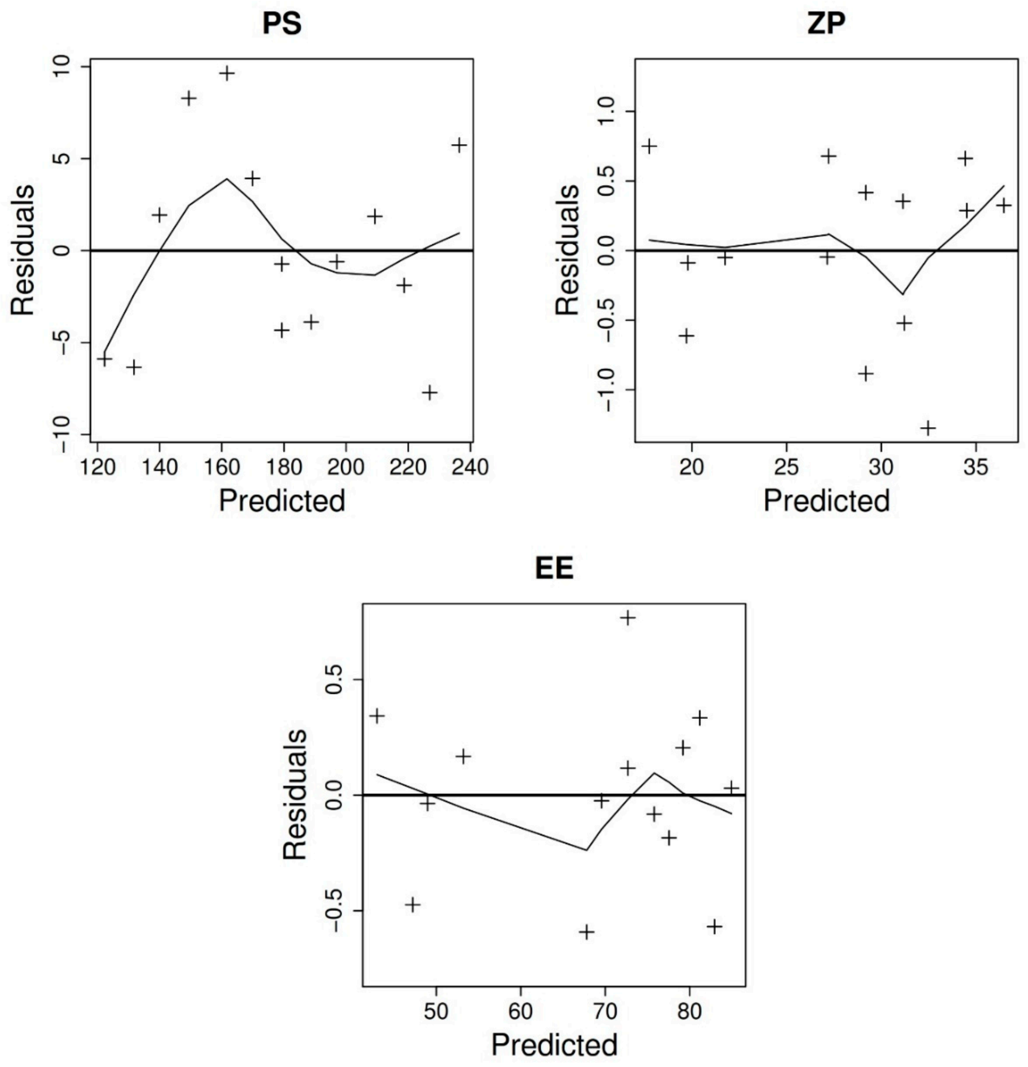

Figure S3. Plot of residual error versus model predicted responses.

Table S1. Analysis of variance (ANOVA) of the final models for measured responses.

Parameters	SS	DF	MS	F	*p*-value	Parameters	SS	DF	MS	F	*p*-value
**PS**						**ZP**					
**Model**	16727.44	3	5575.81	137.16	< 0.0001	**Model**	0.7118	4	0.1779	259.58	< 0.0001
X_1	135.30	1	135.30	3.33	0.0912	X_1	0.0407	1	0.0407	59.39	< 0.0001
X_2	15120.61	1	15120.61	371.96	< 0.0001	X_2	0.6207	1	0.6207	905.47	< 0.0001
X_3	1471.53	1	1471.53	36.20	< 0.0001	X_3	0.0001	1	0.0001	0.1206	0.7344
**Residual**	528.47	13	40.65			X_2^2	0.0503	1	0.0503	73.35	< 0.0001
Lack of Fit	444.75	9	49.42	2.36	0.2118	**Residual**	0.0082	12	0.0007		
Pure Error	83.72	4	20.93			Lack of Fit	0.0043	8	0.0005	0.5478	0.7827
**Cor Total**	17255.90	16				Pure Error	0.0039	4	0.0010		
						**Cor Total**	0.7200	16			

Parameters	SS	DF	MS	F	p-value
**EE**					
**Model**	2683.58	5	536.72	699.43	< 0.0001
X_1	6.14	1	6.14	8.00	0.0164
X_2	127.68	1	127.68	166.39	< 0.0001
X_3	2308.26	1	2308.26	3008.03	< 0.0001
$X_2 X_3$	5.00	1	5.00	6.51	0.0269
X_3^2	236.50	1	236.50	308.20	< 0.0001
**Residual**	8.44	11	0.7674		
Lack of Fit	1.11	7	0.1590	0.0868	0.9966
Pure Error	7.33	4	1.83		
**Cor Total**	2692.02	16			
